# Improving Farmed Juvenile Gilthead Seabream (*Sparus aurata*) Stress Response to Marine Heatwaves and Vibriosis Through Seaweed-Based Dietary Modulation

**DOI:** 10.3390/ani15131970

**Published:** 2025-07-04

**Authors:** Alícia Pereira, Isa Marmelo, Tomás Chainho, Daniel Bolotas, Marta Dias, Rui Cereja, Marisa Barata, Pedro Pousão-Ferreira, Elsa F. Vieira, Cristina Delerue-Matos, Mário S. Diniz, António Marques, Ana Luísa Maulvault

**Affiliations:** 1 CIIMAR—Interdisciplinary Centre of Marine and Environmental Research, University of Porto, Terminal de Cruzeiros do Porto de Leixões, Avenida General Norton de Matos s/n, 4450-208 Matosinhos, Portugal; isa.marmelo@ipma.pt (I.M.); mdias@ciimar.up.pt (M.D.); amarques@ipma.pt (A.M.); 2 IPMA DivAV—Division of Aquaculture, Upgrading and Biosprospection, Portuguese Institute for the Sea and Atmosphere, 1495-165 Algés, Portugal; tomas.chainho@ipma.pt (T.C.); daniel.bolotas@ipma.pt (D.B.); rui.cereja@ipma.pt (R.C.); 3 UCIBIO—Applied Molecular Biosciences Unit, NOVA School of Science and Technology, NOVA University of Lisbon, 2829-516 Caparica, Portugal; mesd@fct.unl.pt; 4 MARE, Marine and Environmental Sciences Centre, Faculty of Sciences, University of Lisbon (FCUL), 1749-016 Lisbon, Portugal; 5 IPMA-EPPO—Aquaculture Research Station, Portuguese Institute for the Sea and Atmosphere, 8700-194 Olhão, Portugal; mbarata@ipma.pt (M.B.); pedro.pousao@ipma.pt (P.P.-F.); 6 REQUIMTE/LAQV—Network of Chemistry and Technology/Associated Laboratory for Green Chemistry, ISEP/IPP—Polytechnic of Porto—School of Engineering, 4249-015 Porto, Portugal; elsa.vieira@graq.isep.ipp.pt (E.F.V.); cmm@isep.ipp.pt (C.D.-M.); 7 Associate Laboratory i4HB—Institute for Health and Bioeconomy, NOVA School of Science and Technology, NOVA University of Lisbon, 2829-516 Caparica, Portugal

**Keywords:** aquaculture, climate change, *Laminaria digitata*, macroalgae, *Vibrio harveyi*, functional feeds, disease outbreaks, antioxidant response, plasma biochemistry

## Abstract

Marine heatwaves (MHWs) are an emerging challenge for aquaculture, increasing the frequency and severity of disease outbreaks in farmed fish, weakening immunocompetence and compromising overall health and survival. As climate change stressors intensify, there is an urgent need for cost-effective and environmentally friendly strategies to enhance fish resilience. This study investigated the efficacy of *Laminaria digitata*, a brown macroalga, included in aquafeeds as powder (0.3% and 1.5%) or extract (0.3%) in improving the stress response of juvenile gilthead seabream (*Sparus aurata*) co-exposed to *Vibrio harveyi* infection during a category III MHW event. Under control conditions, *L. digitata* supplementation maintained or improved fish growth performance and overall well-being. After MHW exposure, the 1.5% powdered and 0.3% extract diets were more effective in mitigating thermal stress, reducing liver oxidative stress and lowering plasma cortisol levels. In infected fish, these diets improved resistance to *V. harveyi*, with reduced cortisol and alanine aminotransferase concentrations indicating hepatoprotective properties, and lower lipid peroxidation and decreased antioxidant enzyme activities reflecting an amplified capacity to counteract oxidative stress induced by inflammation. Overall, *L. digitata* is a promising aquafeed supplement, with the 1.5% powdered form offering a cost-effective alternative to the extract without compromising efficacy.

## Simple Summary

Extreme weather events, like marine heatwaves, are putting pressure on aquaculture by increasing the number and severity of disease outbreaks. These events can weaken farmed fish, making them more vulnerable to illness and reducing their chances of survival. This study explored the effects of adding a brown seaweed, *Laminaria digitata*, to fish feed on the ability of juvenile gilthead seabream (*Sparus aurata*) to cope with high temperatures and bacterial infection. Two forms of the seaweed, powder and extract, were included in the diets at different levels. The results showed that fish fed with seaweed-supplemented diets maintained healthy growth and exhibited reduced stress and liver damage, along with improved resistance to thermal and disease stress. Among the tested diets, the 1.5% powdered form proved to be a cost-effective option for enhancing fish resilience to environmental stressors linked to climate change.

## 1. Introduction

The occurrence of disease outbreaks is a major bottleneck to aquaculture’s sustainable development, compromising animal welfare, seafood security and the economic feasibility of the sector. The stress inherent to captivity, together with high stocking densities and the use of cost-effective diets rich in cereals and vegetable oils and meals, creates the perfect setting to depress animals’ immunocompetence and facilitate the spread of disease outbreaks [1]. As a result, fish farms often resort to antibiotics and chemotherapeutants to control and treat bacterial infections, a practice that not only promotes the incidence of drug-resistant pathogens, such as *Aeromonas salmonicida* and *Edwardsiella tarda* [2], but is also negative due to its environmental and public health impacts [3]. These include the presence of antimicrobial residues that can persist in farmed fish tissues, which can affect consumer safety and compromise the quality of aquaculture products, as well as the discharge of these substances into the aquatic environment, posing risks to non-target organisms and contributing to the spread of antimicrobial resistance [4,5,6]. Climate change is having significant impacts on aquaculture, particularly through the increased occurrence and intensity of extreme weather events, like marine heatwaves (MHWs). These events involve unusually elevated temperatures that can last from days to several months and are especially problematic as they lead to wide-ranging ecological and socioeconomic consequences [7,8]. The sudden exposure to temperatures outside species’ optimal thermal window that takes place during MHWs constitutes an additional physiological challenge to farmed fish, reducing their resilience to captivity-related stressors, namely, disease [9,10]. Indeed, rising temperatures have often been associated with an increased prevalence of disease outbreaks, with severe economic impacts on the aquaculture industry and potential risks to human health. When these rising temperatures are combined with high stocking densities typically occurring in intensive fish production systems, such as marine offshore and coastal aquaculture, the frequency and severity of disease outbreaks are expected to increase even further [11]. *Vibrio* spp., commonly found in tropical and temperate marine environments, are among the primary bacterial pathogens affecting the fish farming industry [12]. Among this genus, *Vibrio harveyi* has recently stood out as one of the most frequently detected agents causative of acute mortality in Mediterranean fish aquaculture [13,14]. This pathogen has been associated with several opportunistic infections in fish, including various cases of infectious necrotizing enteritis, which is characterized by redness of the anal region, abdominal swelling with fluid buildup, inflammation of the anterior intestine and necrosis of the posterior intestine [15]. Disease outbreaks prompted by *V. harveyi* show a distinct seasonal pattern, with higher incidences occurring when water temperatures rise above 20 °C [16,17]. Additionally, increased prevalence of *V. harveyi* is believed to facilitate co-infection with other bacterial pathogens [18]. To manage the challenges posed by disease outbreaks and climate change-related stressors in environmentally sustainable ways, the use of functional feeds with beneficial effects has become a priority area in aquaculture research [4].

It is well established that proper nutrition plays a crucial role in fishes’ overall performance and disease resistance, as diet can influence the growth performance and modulate innate immune responses [19,20]. Furthermore, dietary modulation strategies, such as supplementation with functional ingredients/additives, have demonstrated potential in mitigating the effects of heatwave-induced stress in teleosts [21,22]. In this sense, seaweeds have significant potential as functional ingredients due to their richness in bioactive compounds (e.g., alginates, agar, β-glucan, carrageenans, fucoidan, polyphenols and carotenoids) [23,24], with beneficial effects in both animals and humans [25,26,27,28]. However, while there is considerable information available on the nutritional and functional properties of red and green macroalgae species, research on brown macroalgaes’ potential as an alternative feed ingredient remains limited [24]. The brown macroalga *Laminaria digitata* is especially noteworthy in this regard, as it is rich in bioactive compounds, including polysaccharides (laminarin), phlorotannins and minerals, being suitable as a functional food ingredient for animal feeds [29]. To date, a limited number of studies have investigated the effects of incorporating *L. digitata* into the diets of farmed fish, focusing on aspects such as overall growth performance and immune and antioxidant responses (e.g., [24,28,30,31,32]). Noteworthy, even fewer studies have explored its potential in fish reared under sub-optimal conditions, such as disease outbreaks and/or MHW-induced stress. Therefore, further research is essential to fully explore the potential of *L. digitata* as a functional feed ingredient (including the most effective doses and inclusion form, i.e., dried product or extract) and to validate its effectiveness as a sustainable strategy for mitigating the adverse effects of different environmental stressors.

In this context, the current study aimed to investigate the effectiveness of aquafeeds supplemented with *L. digitata* (as whole dried and extract, at different inclusion percentages) in improving the stress response and resilience of juvenile gilthead seabream (*Sparus aurata*), when co-exposed to an infection with *Vibrio harveyi* during a category III MHW event. To this end, a multi-biomarker approach was employed, integrating different endpoints related to growth performance, plasma biochemistry and antioxidant enzyme activities in the liver.

## 2. Materials and Methods

### 2.1. Experimental Diets

Brown macroalgae *L. digitata* were collected from Parc Naturel Marin d’Irose (West coast of Brittany, France) and transported to Algaia’s factory (Lannilis, France), where 10 kg of the harvested biomass (corresponding to the gametophyte life stage) were selected, milled and dried using bench-top fluid-bed technology (TG200, Retsch, Haan, Germany). Dried samples were ground through a 0.75 mm stainless steel sieve. The *L. digitata* extract was obtained using Subcritical Water Extraction (SWE), in a 400 mL Parr Reactor (Series 4560 high-pressure mini-reactors, Parr Instrument Company, Moline, IL, USA), equipped with a Parr Reactor Controller (Series 4848, Parr Instrument Company, Moline, IL, USA). The extraction was conducted at 40 bar and 180 °C for 50 min, with a 30:1 liquid-to-solid ratio. The sample was agitated at 200 rpm with a four-blade impeller during the process. Following the SWE, the crude extract was filtered through a paper filter (Whatman No. 1), centrifuged at 8000 rpm for 5 min (Sigma 3-30KS, Sigma, Schnelldorf, Germany) and frozen at −80 °C prior to freeze-drying (Teslar, model Cryodos−80, Barcelona, Spain). Finally, extracts were stored at 4 °C, until incorporation in aquafeeds.

Four experimental functional feeds, designed to be isonitrogenous (46.0% crude protein), isolipidic (16.0 crude fat) and isoenergetic (20.5 MJ kg^−1^) were formulated and extruded by SPAROS Lda. (Olhão, Portugal). Initially, all powder ingredients were mixed and ground (<200 microns) using a micropulverizer hammer mill (SH1, Hosokawa-Alpine, Augsburg, Germany). Oils were then added to the powder mixtures, which were humidified with 25% water and agglomerated by a low-shear and low-temperature extrusion process (ITALPLAST, Bertinoro, Italy). The resulting 2.0 mm pellets were subsequently dried in a convection oven at 55 °C for 4 h (OP 750-UF, LTE Scientifics, Oldham, UK). The control diet (CTR) was developed according to the nutritional requirements of juvenile *S. aurata* and did not include any macroalga supplementation (Table 1). Three additional experimental feeds were developed by supplementing the control diet with *L. digitata*, incorporating two powder concentrations of 0.3% (0.3P) and 1.5% (1.5P), and one extract concentration of 0.3% (0.3E), at the expense of wheat meal (Table 1). These inclusion levels were selected based on previous findings indicating no additional functional benefits above 1.5% *L. digitata* powder [24,28]. A lower dose of 0.3% (representing a 5-fold decrease) was included to assess potential dose-dependent effects and to enable a direct comparison between powder and extract.

### 2.2. Fish Rearing and Acclimation Conditions

Gilthead seabream, *S. aurata*, was selected as biological model due to: (i) its ecological and commercial importance in fisheries and aquaculture in the Mediterranean Sea and in the eastern coastal regions of the North Atlantic Ocean [33]; and (ii) it is easy of maintenance in captivity and is suitable to the experimental manipulation, making this specimen a suitable choice for this study. Juvenile specimens with similar morphometry (29.7 ± 4.9 g total weight; 12.2 ± 0.6 cm total length; mean ± standard deviation, *n* = 243) were obtained from the Aquaculture Research Station of the Portuguese Institute for the Sea and Atmosphere (EPPO-IPMA, Olhão, Portugal) and transported to IPMA’s Live Marine Organisms Bioterium (LABVIVOS, Algés, Portugal). Fish were maintained for three weeks in a quarantine system (composed of two tanks with 660 L total capacity each), while being hand-fed twice a day with the CTR feed (2% of their average body weight, bw) and maintained at control rearing conditions: (i) temperature = 21.4 ± 0.5 °C; (ii) pH = 8.0 ± 0.1; (iii) salinity = 35.0 ± 0.5 ‰; (iv) dissolved oxygen (DO) = 7.2 ± 0.2 mg L^−1^; and (v) photoperiod: 14 h light/10 h darkness.

### 2.3. Experimental Design

Following the quarantine period, fish were transplanted to the experimental systems, being randomly distributed in 27 rectangular shape glass recirculation tanks (RAS; 200 L total capacity each). The configuration of each tank—including physical and biological filtration, UV sterilization, temperature regulation and aeration—followed the system setup previously detailed in Pereira et al. [34].

**Figure A1:**
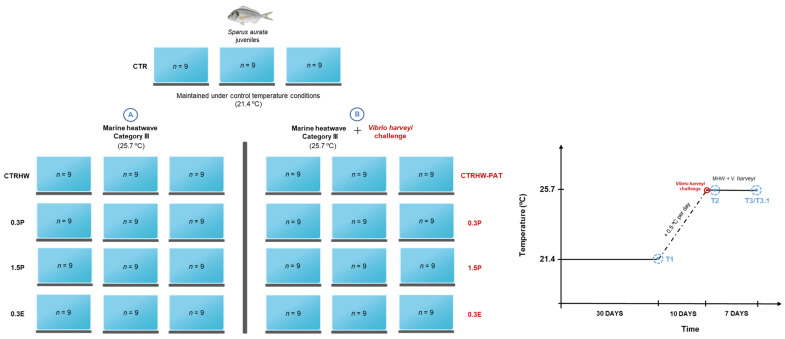
Experimental design representing the treatments not subjected to the *Vibrio harveyi* challenge (**A**) and subjected to *Vibrio harveyi* challenge (**B**) and timeline of supplementation, marine heatwave and *Vibrio harveyi* challenge, including sampling points (T1, T2, T3 and T3.1) for gilthead seabream, *S. aurata*, fed the different experimental diets. Abbreviations: CTR—control feed; CTRHW—control feed exposed to MHW conditions; 0.3P—feed supplemented with 0.3% of dried powdered *L. digitata*; 1.5P—feed supplemented with 1.5% of dried powdered *L. digitata*; 0.3E—feed supplemented with 0.3% of *L. digitata* extract; T1—first sampling, after 30 days of supplementation under optimal conditions (21.4 °C); T2—second sampling, following the 10-day heatwave ramp and 24 h after reaching the MHW peak temperature and the *V. harveyi* challenge; T3—third and final sampling, after 7 days of exposure to the MHW peak temperature alone; T3.1—after 7 days of exposure to a combination of the MHW peak temperature and the *V. harveyi* challenge.

The experimental setup consisted of five treatments, with triplicate tanks assigned to the CTR treatment and six replicate tanks for each of the remaining four treatments (*n* = 9 per tank; Figure A1). The treatments were as follows: (i) CTR—fish fed with the control diet (without any macroalga addition); (ii) CTRHW—fish also fed with the control diet but subjected to a simulated MHW; (iii) 0.3P—fish fed with supplemented feed containing 0.3% of dried powdered *L. digitata* and exposed to a MHW; (iv) 1.5P—fish fed with supplemented feed containing 1.5% of dried powdered *L. digitata* and exposed to a MHW; and (v) 0.3E—fish fed with supplemented feed containing 0.3% of *L. digitata* extract and exposed to a MHW. After seven days of acclimation to the experimental systems, fish from each treatment were hand-fed twice daily (2% bw) with the assigned diets for 30 days under control temperature conditions (21.4 °C; reflecting the average seawater temperature in Mediterranean coastal zones). After this period, a category III MHW (see Section 2.4) was simulated by progressively increasing the water temperature by 0.5 °C per day over 10 days (heatwave ramp; Figure A1) in all treatments, except for CRT treatment, which was maintained under control temperature conditions (21.4 °C) for the entire duration of the trial. After the peak temperature of the MHW was reached (25.7 °C; see Section 2.4), all fish from three tanks (tanks B; Figure A1) in the CTRHW, 0.3P, 1.5P and 0.3E treatments were subjected to a *V. harveyi* challenge (see detailed procedure in Section 2.5), while fish from the three remaining tanks (tanks A; Figure A1) were not exposed to the pathogen. The infected tanks were isolated from the non-infected tanks to prevent horizontal pathogen contamination and to facilitate daily monitoring of animal welfare and survival in each treatment. For seven days (plateau phase of the MHW), fish were exposed either to the MHW peak temperature alone (tanks A; Figure A1) or to a combination of the MHW peak temperature and a *V. harveyi* challenge (tanks B; Figure A1). Throughout this period, animal mortality was monitored and recorded. Humane endpoints were considered for moribund fish and those with ulcerative lesions; these fish were euthanized using an overdose of tricaine methanesulfonate solution (2 g L^−1^; MS-222, Acros Organics, Geel, Belgium) to prevent unnecessary suffering. At the end of the trial, the surviving fish from the pathogen challenge treatments were sacrificed using the same method (MS-222).

Throughout the experiment, key water quality parameters (apart from temperature) were consistently regulated to maintain control conditions: pH at 8.0 ± 0.1, salinity at 35.0 ± 0.5 ‰, DO at 7.2 ± 0.2 mg L^−1^. A controlled photoperiod of 14 h light/10 h darkness was applied. Daily assessments were conducted using a multi-parameter measuring instrument (HI98194, HANNA instruments, Padua, Italy), allowing immediate adjustments when necessary to ensure parameters stability. Ammonia (NH_3_/NH_4_), nitrites (NO_2_^−^), and nitrates (NO_3_^−^) levels were monitored weekly with colorimetric kits (Salifert, Duiven, The Netherlands), keeping nitrates below 50 mg L^−1^. Routine maintenance included the daily removal of fish feces and a 25% water exchange in each tank.

### 2.4. MHW Modulation

Sea surface temperature (SST) data were collected from a Mediterranean area in the Aegean Sea, situated between Greece and Turkey (coordinates: 38°33′10.9″ N 25°02′41.4″ E, 38°06′12.0″ N 25°03′53.0″ E). The dataset, sourced from the National Oceanic and Atmospheric Administration (NOAA) Climate Data Record (CDR), spans 30 years, covering the period from 1993 to 2023.

To assess the intensity, onset and offset rates and duration of MHW events, the “heatwaveR” package [35] within R software version 4.4.2 (R Core Team) was employed, adhering to the standardized MHW definitions by Hobday et al. [36]. The “heatwaveR” package was also used to develop the Yearly Temperature Model (YTM), which provides the average daily SST expected for each day of the year. This model enabled the calculation of the temperature thresholds required for the MHW classification. In this study, the MHW threshold was established at 1.424 °C above the YTM baseline. A baseline SST of 21.4 °C, reflecting the average temperature on June 16th (near the beginning of summer), was chosen. A Category III MHW was simulated, with a peak temperature reaching 25.7 °C, calculated by adding three times the threshold value to the baseline (21.4 °C + (3 × 1.424 °C)).

### 2.5. V. harveyi Challenge

For the pathogen challenge, a *V. harveyi* strain isolated from seabream production (LD50: 4.9 × 10^9^ CFU mL^−1^) and kindly provided from the Culture Collection of the Aquaculture Research Station of the Portuguese Institute for the Sea and Atmosphere (EPPO-IPMA, Olhão, Portugal) was used. The bacterial strain was cultured in tryptic soy broth (TSB, Sigma-Aldrich, Deutschland, Germany) at 24 °C for 48 h with continuous shaking at 160–180 rpm, using the stock strains stored in 25% (*v*/*v*) glycerol at −80 °C. Exponentially growing bacteria were collected by centrifugation at 3000 rpm for 10 min and resuspended in sterile phosphate-buffered saline (PBS; 137 mM NaCl, 2.7 mM KCl, 10 mM Na_2_HPO_4_, 1.8 mM KH_2_PO_4_; Sigma-Aldrich, St. Louis, MO, USA). The suspension was then adjusted, under sterile conditions, to a final concentration of 0.49 × 10^9^ CFU mL^−1^ to prepare the bacterial inoculum for intraperitoneal injection.

Prior to injection, fish were anesthetized by immersion in a MS-222 solution at 0.15 g L^−1^, buffered with sodium bicarbonate (NaHCO_3_, Sigma-Aldrich, St. Louis, MO, USA). Under aseptic conditions, they were intraperitoneally injected with 100 µL of *V. harveyi* suspension (0.49 × 10^9^ CFU mL^−1^). Fish from the non-infected treatments (tanks A; Figure A1) were instead injected with 100 µL of sterile PBS. After the procedures, fish were allowed to recover in aerated seawater before returning to their respective experimental tanks.

### 2.6. Sampling

Fish were sampled at the following sampling points: (i) after 30 days of supplementation under control temperature conditions (T1); (ii) following the 10-day heatwave ramp and 24 h after reaching the MHW peak temperature and *V. harveyi* challenge (T2); (iii) after 7 days of exposure to the MHW peak temperature alone (T3) or after 7 days of exposure to a combination of the MHW peak temperature and a *V. harveyi* challenge (T3.1; Figure A1). Each sampling point resulted in the random collection of six fish per treatment (i.e., two fish from each replicate tanks). At T2, only fish from the pathogen-challenged treatments (tanks B; Figure A1) and the CTRHW treatment were sampled. To maintain consistent culture density and eliminate such effect on fish, an equal number of fish were removed from the remaining treatments (tanks A; Figure A1). Fish were not fed for 24 h prior to sampling. Animals were randomly collected from each tank and anesthetized by immersion in an overdosed MS-222 solution (2 g L^−1^), buffered with sodium bicarbonate. Once anesthetized, fish were measured (total length, TL) and weighed (W). Peripheral blood was then collected from the caudal vein using heparinized syringes and transferred into 1.5 mL microtubes containing 20 µL of heparin (3000 U mL^−1^ in 0.9% NaCl, Sigma-Aldrich, St. Louis, MO, USA). The blood samples were centrifuged at 10,000× *g* for 10 min at 4 °C (Fisher Scientific AccuSpin Micro 17R Centrifuge, Schwerte, Germany) to separate plasma for metabolites analysis, and plasma samples were stored at −80 °C for later analysis of plasma cortisol levels. Subsequently, fish were euthanized by cervical cut, and approximately 100 mg of liver tissue was collected. Liver weight was recorded to calculate the hepatosomatic index (HSI). Tissue samples were frozen and stored at −80 °C for later biochemical biomarker analyses.

### 2.7. Growth Performance Calculations

Fish growth performance and body conditions indexes were calculated based on TL and W, with the following indices calculated:

Condition factor (K) = BW (g)/TL^3^ (cm) × 100 (1)

Hepatosomatic index (HSI, %) = liver weight (g)/BW (g) × 100(2)

Weight gain (GW, %) = (final BW (g) − initial BW (g))/initial BW (g) × 100(3)

Specific growth rate (SGR, % day^−1^) = [Ln(final BW) − Ln (initial BW)]/days × 100 (4)

Feed efficiency was evaluated by calculating the feed conversion ratio (FCR):

FCR = dry feed supplied/fish wet weight gained(5)

### 2.8. Plasma Biochemistry Parameters

Plasma analyses were performed immediately after blood sampling (see Section 2.6). Plasma glucose (GLU), total protein (TP), blood urea nitrogen (BUN), phosphate (PHOS), cholesterol (CHOL), alkaline phosphatase (ALP), alanine aminotransferase (ALT), calcium (CA), globulin (GLOB) and amylase (AMY) were measured using 70 µL of plasma with a Samsung PT10V chemistry analyzer (Samsung Electronics, Suwon, Republic of Korea).

Plasma cortisol concentrations were determined using a colorimetric competitive enzyme immunoassay (ELISA) kit (ADI-900-701, Enzo Life Sciences, Inc., Farmingdale, NY, USA), following the manufacturers protocol. Cortisol levels were calculated using a standard curve established from cortisol standards included in the kit. Samples and standards absorbance was read at 405 nm.

### 2.9. Oxidative Stress Response

Liver samples were homogenized under ice-cold conditions with 1 mL of PBS (pH 7.4; 137 mM NaCl, 2.7 mM KCl, 10 mM Na_2_HPO_4_, 1.8 mM KH_2_PO_4_; Sigma-Aldrich, St. Louis, MO, USA) using an Ultra-Turrax device (T10 basic, Ika, Baden-Württemberg, Germany). The crude homogenates were centrifuged in 1.5 mL microtubes at 10,000× *g* and 4 °C for 10 min and the supernatants were collected into new microtubes and stored at −80 °C until biomarker analyses were performed. These supernatants were used to determine the following oxidative stress biomarkers: catalase (CAT) activity, glutathione S-transferase (GST) activity, superoxide dismutase (SOD) activity and lipid peroxidation (LPO). Detailedprotocols are described in Marmelo et al. [24]. Biomarker results for all samples were normalized by dividing by total protein content (expressed in mg of protein; except for SOD, % inhibition), determined using the Bradford assay [37]. All assays were adapted for use in 96-well microplates and performed using a microplate reader (Thermo Scientific Multiskan GO 1510, Waltham, MA, USA). Each sample was analyzed in triplicate or more, employing reagents and standards of analysis grade or higher.

### 2.10. Statistical Analysis

To evaluate the effects of diet under control temperature conditions and after exposure to MHW conditions (T1 vs. T3), as well as following combined MHW exposure with a *V. harveyi* challenge (T2 vs. T3.1), growth performance parameters, plasma biochemistry and biomarker levels results were analyzed using a two-way ANOVA, considering both diet and sampling point as factors. For comparisons after exposure to the MHW (T3) data was analyzed using a one-way ANOVA. When significant differences were identified (*p* < 0.05), multiple comparisons were conducted using Tukey HSD post hoc test. Prior to ANOVA analyses, data were checked for normality and homogeneity of variance using Kolmogorov–Smirnov and Levene tests, respectively. All statistical analyses were conducted using STATISTICATM software (Version 7.0, StatSoft Inc., Tulsa, OK, USA).

## 3. Results

### 3.1. Growth Performance

Results on body condition indexes and growth performance parameters are presented in Table 2, comparing outcomes under control temperature conditions with those following exposure to the peak temperature of the MHW alone (T1 vs. T3), and after 24 h and 7 days of combined exposure to the MHW peak temperature and a *V. harveyi* challenge (T2 vs. T3.1). The two-way ANOVA *p*-values for the interactions between the two factors (treatments and sampling point) for each parameter evaluated are available in the Supplementary Materials (Table S1).

At T1, HSI values were significantly lower in 0.3P and 1.5P treatments compared to CTR (*p* < 0.001). At T3, fish from the CTRHW treatment revealed lower HSI values compared to fish from the CTR treatment (*p* < 0.001). At T1, fish fed the 0.3P and 0.3E diets showed significantly higher SGR compared to the CTR treatment (*p* < 0.001). However, no significant differences were observed between the CTR and CTRHW treatments in SGR at T3 (*p* > 0.05), though fish fed the 1.5P diet showed significantly higher SGR values compared to CTRHW fish (*p* < 0.001). When comparing sampling days (T1 vs. T3) within the same treatment, fish fed the 0.3E diet showed significantly lower HSI values at T3 (*p* < 0.001), while SGR values were significantly lower in 0.3P and 0.3E treatments at T3 (*p* < 0.001). Additionally, FCR values were significantly higher across all treatments in T3 (*p* < 0.001).

After 24 h (T2) and 7 days (T3.1) of exposure to the combined effects of the MHW peak temperature and the *V. harveyi* challenge, fish fed with the 0.3P diet showed significantly lower K compared to the CTRHW-PAT treatment at T3.1 (*p* < 0.001). At T2, HSI values were significantly higher in the CTRHW-PAT treatment compared to the CTRHW treatment (*p* < 0.001), with no differences observed between the CTRHW-PAT and supplemented treatments (*p* > 0.05). Similarly, at T3.1, the CTRHW-PAT treatment showed significantly higher HSI values than the CTRHW treatment (*p* < 0.001). However, fish fed the *L. digitata*-supplemented diets showed significantly lower HSI values compared to CTRHW-PAT treatment (*p* < 0.001). In terms of SGR, fish from the CTRHW-PAT treatment demonstrated significantly higher values than those in the CTRHW treatment at T2 (*p* < 0.001). Conversely, at T3.1, no differences in SGR were found between the CTRHW and CTRHW-PAT treatments (*p* > 0.05). However, fish fed the 1.5P diet showed a significantly higher SGR compared to the CTRHW-PAT treatment (*p* = 0.018). As for comparisons between T2 and T3.1 results, fish fed the 0.3P diet exhibited a lower K value at T3.1 (*p* = 0.006), while HSI values were significantly lower in the 1.5P treatment (*p* < 0.001). SGR was significantly lower at T3.1 in the CTRHW-PAT, 0.3P and 0.3E treatments (*p* < 0.001). Lastly, FCR values were significantly higher in fish fed the 0.3P diet at T3.1 (*p* < 0.001).

### 3.2. Plasma Biochemistry

Table 3 provides a summary of the plasma biochemistry results (two-way ANOVA *p*-values for the interactions between the treatment and sampling point for each metabolite evaluated are available in the Supplementary Materials; Table S1). At T1 and T3, GLU levels were significantly higher in the *L. digitata*-supplemented treatments compared to the CTR and the CTRHW treatment, respectively (*p* = 0.001 for 0.3P, *p* = 0.007 for 1.5P and *p* = 0.001 for 0.3E at T1; *p* = 0.035 for 0.3P, *p* = 0.001 for 1.5P and *p* = 0.040 for 0.3E at T3). At T1, fish fed with the 1.5P diet showed significantly decreased TP levels compared to the CTR (*p* = 0.044). However, at T3, only fish fed with the 0.3P diet revealed significantly lower levels of TP compared to CTRHW (*p* = 0.021). Significant differences in BUN levels were observed only at T3, where fish fed the 0.3P and 1.5P diets had significantly higher levels compared to the CTRHW treatment (*p* = 0.041 and *p* = 0.049, respectively). ALT values were significantly lower in the 0.3P and 1.5P treatments compared to CTR at T1 (*p* = 0.023 and *p* = 0.008, respectively). Conversely, at T3, these treatments showed significantly higher ALT levels compared to the CTRHW (*p* = 0.042 and *p* = 0.004, respectively), while the 0.3E treatment revealed significantly lower levels (*p* = 0.001). CA levels were significantly higher in fish fed the 0.3P diet compared to CTR at T1 (*p* < 0.001). However, at T3, all *L. digitata*-supplemented treatments showed significantly higher levels of CA in comparison with the CTRHW treatment (*p* = 0.004 for 0.3P, *p* = 0.001 for 1.5P and *p* = 0.016 for 0.3E). Regarding ALP, lower levels were observed in the 1.5P and 0.3E treatments compared to CTR at T1 (*p* = 0.013 and *p* = 0.015, respectively). Nevertheless, at T3 the 1.5P treatment revealed significantly higher ALP levels compared to CTRHW (*p* = 0.004). CHOL levels showed significant differences only at T3, with fish fed the 0.3E diet revealing higher levels compared to the CTRHW (*p* = 0.005). When comparing sampling days (T1 vs. T3) within the same treatment, fish fed the 0.3P diet exhibited higher GLU levels at T3 (*p* = 0.012), while those fed the 0.3E diet showed increased TP levels (*p* = 0.024). ALT levels were significantly lower at T3 in the CTR and 0.3E treatments (*p* < 0.001 and *p* = 0.008, respectively). Fish fed with the 0.3P diet demonstrated reduced CA levels at T3 (*p* = 0.006), whereas those on the 1.5P diet revealed CA higher levels (*p* = 0.043). Additionally, ALP levels were significantly higher in the 1.5P treatment at T3 (*p* < 0.001). Finally, cortisol levels were significantly higher in the 0.3P and 0.3E at T3 (*p* < 0.001 and *p* = 0.007, respectively). After 7 days (T3.1) of exposure to the combined stress of the MHW peak temperature and the *V. harveyi* challenge, significant differences in GLU levels were observed between CTRHW-PAT and CTRHW treatments (*p* < 0.001), as well as between CTRHW-PAT treatment and fish fed the 0.3P and 1.5P diets (*p* = 0.003 and *p* = 0.025, respectively). At T2, significantly lower levels of BUN were observed in the CTRHW-PAT treatment compared to CTRHW (*p* < 0.001). In contrast, PHOS levels were significantly reduced in the CTRHW-PAT treatment at both T2 and T3.1 (*p* = 0.003 and *p* = 0.043, respectively). ALT levels were significantly elevated in the CTRHW-PAT fish compared to CTRHW (*p* < 0.001) at T2. In contrast, fish fed the 1.5P and 0.3E feeds showed significantly lower ALT levels compared to CTRHW-PAT (*p* < 0.001 and *p* = 0.009, respectively). At T3.1, fish fed the 0.3P diet exhibited higher ALT levels than CTRHW-PAT fish (*p* < 0.001). ALP levels were significantly elevated in the CTRHW-PAT compared to CTRHW at both T2 and T3.1 (*p* < 0.001 and *p* = 0.039, respectively). However, at T3.1, fish fed with the 0.3P diet exhibited higher ALP levels compared to CTRHW-PAT (*p* = 0.001). Regarding AMY levels, 0.3P treatment revealed significantly higher levels compared to CTRHW-PAT at T3.1 (*p* = 0.007). At T2, cortisol levels were significantly elevated in the CTRHW-PAT treatment in comparison with the CTRHW treatment (*p* < 0.001); however, fish fed with 1.5% powder and 0.3% extract showed reduced cortisol levels relative to CTRHW-PAT, with lower levels being observed in the 0.3E treatment (*p* < 0.001). At T3.1, cortisol levels were significantly lower in the supplemented treatments compared to CTRHW-PAT (*p* < 0.05). Regarding the comparisons between T2 and T3.1 results, significant changes were observed across several parameters. GLU levels significantly increased in the CTRHW-PAT and 0.3E treatments at T3.1 (*p* < 0.001 and *p* = 0.005, respectively). At the same sampling point, BUN levels were significantly lower in the CTRHW but higher in the 1.5P treatment (*p* < 0.001 and *p* = 0.009). PHOS levels were decreased significantly in both CTRHW-PAT and 0.3E treatments at T3.1 (*p* = 0.006 and *p* = 0.049), while ALT levels were significantly decreased in both CTRHW-PAT treatment and all supplemented treatments at T3.1 (*p* < 0.001). ALP levels were significantly lower in the CTRHW, CTRHW-PAT and 1.5P (*p* < 0.001). Lastly, cortisol levels were significantly lower in the 0.3P and 1.5P treatments at T3.1 (*p* < 0.001 and *p* = 0.016).

### 3.3. Oxidative Stress

**Figure 1:**
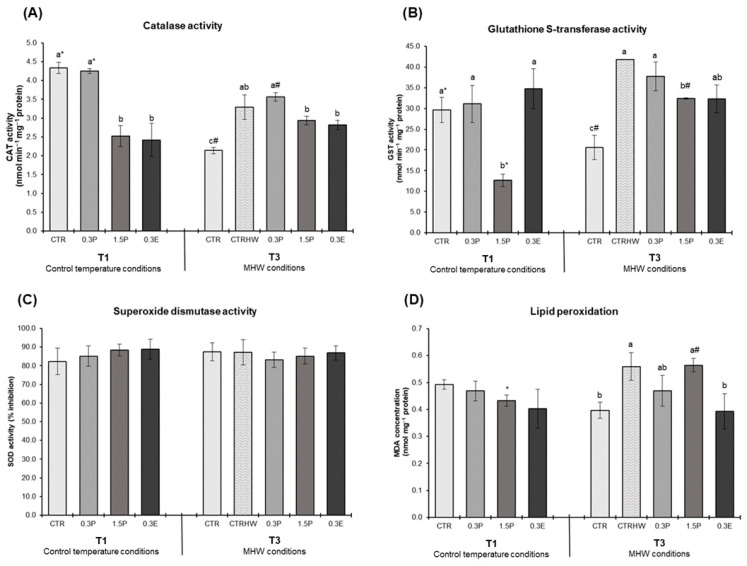
Oxidative stress biomarkers in the liver of *S. aurata* fed the experimental diets after 30 days of supplementation control temperature conditions (T1) and after 7 days of exposure to the MHW peak temperature alone (T3) (mean ± SD; *n* = 6). (**A**)—catalase (CAT) activity (nmol min^−1^ mg^−1^ protein); (**B**)—glutathione S-transferase (GST) activity (nmol min^−1^ mg^−1^ protein); (**C**)—superoxide dismutase (SOD) activity (% inhibition); and (**D**) lipid peroxidation (LPO, expressed as MDA concentration, nmol mg^−1^ protein). Different letters denote significant differences between treatments on the same sampling time and different symbols (* and #) indicate significant differences between sampling times (T1 and T3) for the same treatment (*p* < 0.05). The absence of letters or symbols indicates no statistical difference. Abbreviations: CTR—control feed; CTRHW—control feed exposed to MHW conditions; 0.3P—feed supplemented with 0.3% of dried powdered *L. digitata*; 1.5P—feed supplemented with 1.5% of dried powdered *L. digitata*; 0.3E—feed supplemented with 0.3% of *L. digitata* extract; MDA—malondialdehyde.

Figure 1 presents fish antioxidant enzyme activities (CAT, GST and SOD) and lipid peroxidation (LPO) levels in the liver of juvenile *S. aurata* after 30 days of supplementation under control temperature conditions (T1) and following 7 days of exposure to the peak temperature of the MHW (T3) (two-way ANOVA *p*-values for the interactions between treatments and sampling points for each biomarker evaluated can be consulted in the Supplementary Materials; Table S1). At T1, CAT activity was significantly lower in fish fed the 1.5P and 0.3E diets compared to the CTR (*p* < 0.001; Figure 1A). At T3, CAT activity in the CTRHW treatment was higher than in the CTR (*p* < 0.001; Figure 1A). GST activity was lower in the 1.5P treatment in relation to CTR at T1 (*p* < 0.001; Figure 1B). At T3, GST activity levels were significantly elevated in the CTRHW compared to the CTR treatment (*p* < 0.001), while fish fed the 1.5P and 0.3E diets exhibited significantly lower GST levels compared to the CTRHW (*p* = 0.007; Figure 1B). At T3, LPO levels were significantly higher in fish from the CTRHW compared to the CTR treatment (*p* = 0.014), with only fish fed with the 0.3E diet showing significantly lower LPO values than those in the CTRHW (*p* = 0.012: Figure 1D). When comparing sampling points (T1 vs. T3) within the same treatment, CAT activity in the CTR and 0.3P treatments significantly decreased at T3 (*p* < 0.001 and *p* = 0.016, respectively; Figure 1A). In the 1.5P treatment at T3, GST activity and LPO levels significantly increased (*p* < 0.001 and *p* = 0.040, respectively; Figure 1B,D).

**Figure 2:**
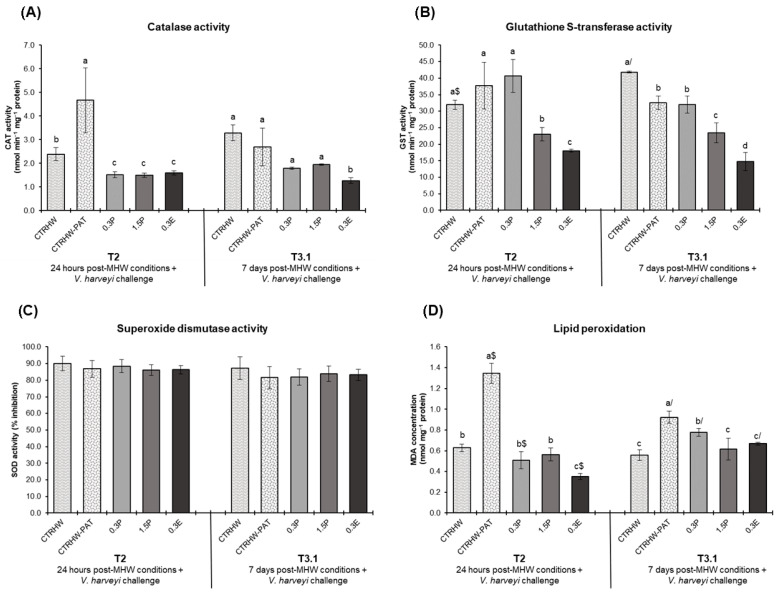
Oxidative stress biomarkers in the liver of *S. aurata* fed the experimental diets following 24 h after exposure to the MHW peak temperature and *V. harveyi* challenge (T2) and after 7 days of exposure to a combination of the MHW peak temperature and the *V. harveyi* challenge (T3.1) (mean ± SD; *n* = 6). (**A**)—catalase (CAT) activity (nmol min^−1^ mg^−1^ protein); (**B**)—glutathione S-transferase (GST) activity (nmol min^−1^ mg^−1^ protein); (**C**)—superoxide dismutase (SOD) activity (% inhibition); and (**D**)—lipid peroxidation (LPO, expressed as MDA concentration, nmol mg^−1^ protein). Different letters denote significant differences between treatments on the same sampling time and different symbols ($ and /) indicate significant differences between sampling times (T2 and T3.1) for the same treatment (*p* < 0.05). The absence of letters or symbols indicates no statistical difference. Abbreviations: CTR—control feed; CTRHW—control feed exposed to MHW conditions; 0.3P—feed supplemented with 0.3% of dried powdered *L. digitata*; 1.5P—feed supplemented with 1.5% of dried powdered *L. digitata*; 0.3E—feed supplemented with 0.3% of *L. digitata* extract; MDA—malondialdehyde.

The antioxidant response of fish exposed to the MHW after 24 h (T2) and 7 days (T3.1) following the *V. harveyi* challenge are presented in Figure 2. At T2, CAT activity showed a significant increase in the CTRHW-PAT treatment compared to CTRHW (*p* = 0.001), while all supplemented treatments exhibited lower CAT activity levels than the CTRHW-PAT (*p* < 0.001; Figure 2A). By T3.1, only fish fed the 0.3E diet showed a significant reduction in CAT activity compared to the CTRHW-PAT (*p* = 0.009; Figure 2A). GST levels at T2 were significantly lower in the 1.5P and 0.3E treatments compared to the CTRHW-PAT (*p* < 0.001; Figure 2B). At T3.1, GST activity in the CTRHW-PAT treatment was significantly reduced compared to the CTRHW (*p* = 0.005), with further reductions observed in the 1.5P and 0.3E treatments relative to CTRHW-PAT (*p* = 0.005 and *p* < 0.001, respectively; Figure 2B). Furthermore, LPO levels at T2 were significantly elevated in the CTRHW-PAT compared to the CTRHW (*p* < 0.001: Figure 2D). However, fish in the supplemented treatments showed reduced LPO levels compared to the CTRHW-PAT, with fish fed the 0.3E diet presenting the lowest value (*p* < 0.001). A similar pattern was observed at T3.1, with the lowest LPO levels recorded in fish fed both 1.5P and 0.3E diets (*p* < 0.001 and *p* = 0.003, respectively; Figure 2D). Comparing sampling points, LPO levels were significantly lower in the CTRHW-PAT and 0.3P (*p* < 0.001 and *p* = 0.002, respectively), while significantly increased in the 0.3E treatment (*p* < 0.001; Figure 2D).

## 4. Discussion

### 4.1. Supplementation Under Control Temperature and MHW Conditions

Optimizing a diet often involves balancing the functional advantages of new ingredients with their potential effects on growth and nutrient utilization efficiency. In this sense, it is important to note that the absence of significant changes (regardless of inclusion percentages) in fish morphometry (TL and W), condition (K) and feed efficiency (FCR) observed in this study indicates that 30 days of supplementation under control temperature conditions with *L. digitata* did not compromise the growth or impaired nutrient utilization in *S. aurata* juveniles. Notably, although there was a lack of statistically significant differences, the consistently high K values (>1), suggest that fish remained well nourished throughout the experimental period. Moreover, the observed significant improvement of SGR in animals fed with 0.3% *L. digitata* (incorporated either as dried seaweed or extract), points to the beneficial effect of this inclusion dose on fish overall performance. Seaweeds contain bioactive compounds, such as phenolics, which have shown conflicting effects on growth performance. High doses of polyphenols may cause anti-nutritional effects, negatively affecting both growth and nutrient utilization efficiency [38]. This could explain why 1.5% of *L. digitata* did not yield proportional improvements. However, it is important to emphasize that the 1.5% inclusion level maintained SGR, K and FCR similar to the control, indicating no adverse effects. Similarly, Ribeiro et al. [30] found that the inclusion of 10% *L. digitata* in *S. aurata* diets did not negatively affect growth performance or feed conversion ratios. However, it is important to note that their study had a longer trial duration (118 days), involved larger fish and used different experimental conditions. Furthermore, Kamunde et al. [39] reported higher SGR in Atlantic salmon (*Salmo salar*) fed diets containing 3% and 10% brown seaweed flakes, prepared with *Laminaria* sp., during a 30-day feeding trial.

The current study also indicated that fish fed diets supplemented with dried powdered *L. digitata*, specifically the 0.3P and 1.5P diets, had lower HSI. This reduction, only observed in fish supplemented with *L. digitata* in the form of dried powder, might be related with the presence of certain bioactive compounds in seaweed powder that may not be bioavailable in the extracts. The extraction process (i.e., sub-critical extraction using water as solvent) might selectively concentrate certain bioactive compounds while removing others that can be crucial for modulating liver metabolism or promoting lipid catabolism. In contrast, the powdered form retains the *L. digitata* full bioactive compounds profile, which may interact synergistically to regulate lipid metabolism, reducing lipids deposition in the liver, ultimately reducing HSI. Yet, the confirmation of this hypothesis can only be achieved through an extensive characterization of *L. digitata* dried powder and sub-critical extract (currently being undertaken in the framework of a complementary study). In accordance with this hypothesis, Vizcaíno et al. [40] also reported lower HSI in juvenile *S. aurata* supplemented with *Gracilaria cornea* and *Ulva rigida*. These authors argued that this reduction (most likely resultant from less fatty liver) was associated with more efficient lipid utilization by fish, diminishing the storage of lipids in the hepatocytes.

Organisms subjected to temperatures beyond their thermal tolerance typically experience a decrease in fitness due to the higher energy demands required to maintain internal homeostasis, which diverts resources that would otherwise support somatic growth under more favorable environmental conditions [32,41]. In this study, although most growth parameters including TL, W, K and FCR remained consistent among treatments, HSI was lower in fish fed the control diet after 7 days of exposure to MHW peak temperature (CTRHW; T3) compared to those reared under control temperature conditions (CTR), indicating a potential adaptive metabolic adjustment. In other words, this reduction may reflect a shift in energy utilization, as high temperatures influence lipid and carbohydrate metabolism [42], possibly favoring energy mobilization to meet the increased metabolic demands, over storage. In terms of SGR values, the significant increase recorded in fish fed the 1.5P diet suggests that this feed formulation may constitute a more balanced nutritional option under stressful conditions. FCR was consistently higher in all treatments following the 7 day exposure to the MHW (T3) compared to control temperature conditions (T1), reflecting a lower feed conversion, likely due to increased metabolic demands and stress induced by elevated temperatures. Nevertheless, the higher FCR did not result in significant reductions in SGR values at T3, suggesting that growth was maintained despite the decreased efficiency in feed utilization under thermal stress.

Plasma biochemical parameters provide information on physiological responses and serve as a functional indicator of the nutritional status and adaptation to a novel dietary ingredient [43,44]. At the end of the supplementation period under control temperature conditions (T1), the inclusion of *L. digitata* significantly influenced the majority of the analyzed plasma parameters. GLU levels reflect changes in the metabolism and energy demand of the organisms [45]. In this study, fish fed *L. digitata*-supplemented diets demonstrated higher GLU concentrations compared to those fed on control diet. These results are in line with a previous study where the inclusion of 8% of *Gracilaria gracilis* in the diet of European seabass (*Dicentrarchus labrax*) increased plasma GLU levels. Curiously, a reduction in TP was observed in fish fed with 1.5% *L. digitata* diet, which could indicate the activation of protein catabolism [46]. This metabolic process can compromise fish’s immune system and result in the degradation of vital proteins, increasing their vulnerability to diseases [47,48,49]. Alterations in hepatic enzyme activity, such as ALT, are recognized as indicators of liver function, with elevated levels reflecting hepatic stress or damage [44,50]. This is particularly relevant, since farmed fish often exhibit histopathological alterations, such as hepatic steatosis (fatty liver), often attributed to diet composition and reduced physical activity under captive conditions [51]. In this study, ALT levels in fish fed the 0.3P and 1.5P diets were significantly lower than those observed in the CTR treatment, suggesting that the inclusion of *L. digitata* provides a hepatoprotective effect, potentially minimizing liver damage [52,53]. This improved liver function may, in turn, result in a more efficient lipid metabolism and reduce unnecessary lipid accumulation, as reflected by the lower HSI values observed in fish fed with these diets. Furthermore, the significant reduction in ALP levels in fish fed with the 1.5% dried powdered *L. digitata* and with 0.3% of *L. digitata* extract diets, along with unchanged BUN levels in fish on the supplemented diets, further reinforces the previous assumption [46]. Together, these findings point to a beneficial role of *L. digitata* in supporting hepatic health and in maintaining metabolic balance. Plasma CA is recognized as a reliable indicator of the secondary phase of stress response in fish and is often used as an indirect marker of changes in plasma cortisol [43]. A significant increase in CA levels was observed in fish fed with 0.3% dried powdered *L. digitata* diet. However, plasma cortisol levels remained consistent between non-supplemented and dried powdered diets, suggesting that these diets did not trigger a cortisol-mediated response.

The seven-day exposure to the MHW peak temperature (T3) modified several plasma biochemistry parameters. Plasma cortisol and glucose levels are the primary metabolites released into the plasma, serving as key indicators of the stress response [54]. In this study, GLU levels were significantly influenced by diets, with higher levels being observed in fish fed with the supplemented diets compared to those fed on the control diet. This may suggest an increased energy demand required to cope with the stress effects, as glucose, an important carbohydrate influencing the bioenergetics of organisms, tends to increase in response to environmental stress [55,56]. In addition, the elevated glucose levels in fish fed the supplemented diets may also reflect the higher availability of polysaccharides derived from *L. digitata*, which could enhance carbohydrate metabolism. In contrast, the cortisol levels of animals fed with diets containing *L. digitata* remained unaltered compared to the CTRHW treatment. The present findings also demonstrated that cholesterol content significantly increased in fish fed with 0.3% extract of *L. digitata* after the exposure to the MHW alone, while fish fed the remaining supplemented diets maintained CHOL levels similar to control treatments. The transfer of cholesterol from the liver to peripheral cells helps to mitigate lipid peroxidation damage caused by heat shock [57]. Therefore, the observed rise in plasma CHOL levels suggests that the inclusion of 0.3% extract of *L. digitata* plays an important role in the protection against liver damage. However, ALT levels were significantly higher in fish fed with diets containing 0.3% and 1.5% dried powdered *L. digitata*, while ALP levels were higher in fish fed with the 1.5P diet. Additionally, BUN levels were elevated in fish fed with both inclusion levels of the dried powdered diets compared to the CTRHW, indicating potential liver stress or damage [43]. Although ALT, ALP and BUN levels were elevated, these changes were not reflected in HSI.

The activity of antioxidant enzymes (CAT, GST and SOD) along with LPO levels were assessed in *S. aurata* liver. Results showed that incorporating *L. digitata* into aquafeeds under control temperature conditions significantly reduced CAT activity, particularly in 1.5P and 0.3E treatments, and GST activity in the 1.5P treatment, while SOD and LPO remained unchanged in all treatments, suggesting an effective maintenance of the redox state and a diminished need to neutralize ROS and lipid peroxides in this tissue. Brown seaweeds, including *L. digitata*, typically have higher antioxidant activity than red and green algae [58], primarily due to their high levels of phlorotannins (phenolic compounds), that play a key role in the protection against oxidative damage [59]. Consequently, it is likely that the antioxidant compounds present in *L. digitata* help to prevent the accumulation of oxidative compounds, such as lipid peroxidation products, thereby reducing the need for antioxidant enzyme activity. These results are consistent with those of a previous study with the same fish and seaweed species, where a reduction in antioxidant enzymes activity and LPO levels in muscle and liver tissues was observed, particularly with lower *L. digitata* inclusion percentages (<3%) [28]. Results also showed that the exposure to a MHW led to enhanced CAT and GST activities in non-supplemented fish compared to those reared under control temperature conditions, highlighting the organism’s efforts to counteract oxidative stress. However, despite the upregulation of these antioxidant defenses, ROS formation was not fully mitigated, resulting in increased LPO levels in non-supplemented fish exposed to the MHW. Regarding the potential of *L. digitata* to prevent the damage caused by MHWs, the diets containing 1.5% dried powdered and 0.3% extract were effective in reducing CAT and GST activities. Additionally, the 0.3% extract diet lowered LPO levels, suggesting that this inclusion level may play a key modulatory role in the antioxidant response under thermal stress conditions. This combined effect, reflected in the reduction in both lipid peroxidation and antioxidant enzyme activity, highlights the potential of *L. digitata* to alleviate oxidative stress. These findings are in line with previous studies [27,34], where the inclusion of *A. taxiformis*, a red seaweed, in juvenile white seabream (*Diplodus sargus*) diets exposed to MHW conditions effectively reduced CAT and GST activities, as well as lipid peroxidation in the muscle, liver and spleen tissues.

### 4.2. Combined Effects of MHW and V. harveyi Challenge

Bacterial challenge tests serve as a robust method for assessing the effects of supplemented diets [1]. After 24 h of combined exposure to the MHW peak temperature and the *V. harveyi* challenge (T2), fish fed with the control diet and exposed to *V. harveyi* (CTRHW-PAT) showed significantly higher HSI values compared to those fed with the control diet without exposure to the bacterial challenge. This increase may indicate a shift in the liver’s role from metabolic adaptation (as observed in T3—MHW exposure alone) to immune and inflammatory activity, reflecting the organism’s effort to counteract the combined stress. It may also indicate pathological liver damage or inflammation triggered by the bacterial infection, especially under the added stress of elevated temperatures. In addition to the elevated HSI values, fish from the CTRHW-PAT treatment also exhibited higher SGR values compared to the CTRHW treatment. The combination of elevated temperatures and the bacterial challenge may have triggered a metabolic shift that inadvertently favored growth, potentially through increased feeding rates or the redirection of energy towards somatic growth. Elevated temperatures, within a tolerable range, are known to increase metabolic activity, feeding rates and digestive efficiency in fish, which can lead to enhanced growth performance [32,60]. In addition, the immune challenge may have acted as a mild stressor, triggering a compensatory response that redirected energy toward maintain homeostasis and supporting growth. Following 7 days of exposure to MHW conditions and the *V. harveyi* challenge (T3.1), a similar pattern to T2 was observed for the CTRHW-PAT treatment regarding HSI. However, fish fed with the *L. digitata* supplemented diets, regardless of the inclusion level or form, revealed significantly lower HSI levels compared to the CTRHW-PAT, suggesting that the supplementation may have alleviated the stress impact on the liver. In terms of SGR values, fish fed with the 1.5P diet revealed a significant increase in SGR, reinforcing the potential positive impact of this inclusion level of *L. digitata* in improving fish growth performance during challenging conditions. Fish fed with the 0.3% dried powdered *L. digitata* diet showed lower K values compared to fish from the CTRHW-PAT treatment. Nevertheless, the values remained considerably high (>1), indicating that the fish were still well nourished.

The *V. harveyi* challenge significantly affected the plasma biochemistry of *S. aurata* juveniles, aligning with previous findings that pathogenic manifestations are reflected in plasma biochemistry profiles [43]. The bacterial challenge induced a pronounced stress response within the first 24 h (T2), as evidenced by a significant increase in plasma cortisol levels in fish fed with the control diet (CTRHW-PAT). This stress response was accompanied by a significant increase in PHOS in fish from the same treatment. Similarly to CA, PHOS is recognized as an effective marker of the secondary phase of stress response in fish and serves as an indirect indicator of changes cortisol levels [43]. Such increase is expected as pathological conditions may alter serum PHOS concentrations [61]. Additionally, fish from the CTRHW-PAT treatment showed increased activities of ALT and ALP, suggesting heightened liver stress or compromised liver function. These findings are further supported by the higher HSI observed in fish from the same treatment. Nevertheless, the dietary inclusion of *L. digitata* demonstrated a protective effect against stress. Fish fed with the 1.5% powder and 0.3% extract supplemented diets showed significantly lower cortisol levels compared to the control fish, with the lowest cortisol concentrations being found in fish fed with the 0.3% extract of *L. digitata*. Furthermore, ALT levels were significantly reduced in fish fed with 1.5% powder and 0.3% extract of *L. digitata*, indicating improved liver health and reduced hepatic stress. After 7 days, PHOS and ALP levels remained significantly elevated in the CTRHW-PAT fish, suggesting that these fish were unable to recover adequately from the combined stress of MHW exposure and *V. harveyi* challenge. Additionally, in contrast to the absence of significant differences in ALT and ALP levels between fish fed with 0.3% dried powdered *L. digitata* diet and those fed with the control diet at T2, fish fed with the 0.3P diet showed significantly higher levels of these markers after 7 days. This pattern suggests that the 0.3P diet may have initially provided some resilience to the combined stress of MHW and bacterial challenge, as evidenced by the similar ALT and ALP after the first 24 h. However, the significantly elevated ALT and ALP levels observed after 7 days indicate that fish eventually experienced physiological stress or damage, as a result of prolonged exposure to these stressors. Despite this, fish fed with the supplemented diets revealed reduced levels of cortisol after 7 days of exposure. However, the pronounced effect of the 0.3E diet observed at T2 appeared to diminish overtime, as cortisol levels in fish fed with this diet became similar to those fed with the 0.3P and 1.5P diets at T3.1. This pattern suggests that while the 0.3% extract diet initially provided rapid stress mitigation benefits, its effectiveness may have declined over time, underscoring the importance of assessing not only the duration and consistency of dietary interventions but also their cost-effectiveness for the aquaculture sector, particularly when comparing the use of extracts versus powdered formulations.

During infections, activated phagocytes produce ROS as part of the immune response, leveraging their microbicidal properties to combat and degrade pathogens [62]. However, when ROS production becomes excessive, it can result in oxidative stress, a phenomenon commonly reported in infection related conditions [63]. In this study, the first 24 h of the *V. harveyi* challenge resulted in significantly increased LPO levels and CAT activity in non-supplemented fish injected with the *V. harveyi* suspension compared to those not exposed to the pathogen. However, the inclusion of *L. digitata* in diets appears to help maintaining the redox balance after infection, as lower LPO levels were detected in infected fish fed *L. digitata* supplemented diets, with the lowest values observed in fish fed with extract diet. Additional evidence of improved detoxifying activity in *S. aurata* was provided by reduced CAT activity across all supplemented diets and by lower GST activity, particularly in fish fed with 1.5% powder and 0.3% extract, with the extract yielding the lowest levels. After 7 days post-infection, ROS formation was still not fully prevented, culminating in increased LPO values in the liver of non-supplemented fish, although these values were lower than those observed at T2. However, fish fed with 1.5% dried powder and 0.3% *L. digitata* extract diets showed lower GST and LPO levels, while those fed with 0.3% extract also exhibited lower CAT activity. Interestingly, similar to the trend observed in cortisol levels, the strong antioxidant effect of the 0.3E diet observed at T2 seemed to weaken over time, with LPO levels in fish fed with this diet aligning with those fed with the 1.5P treatment at T3.1. This further reinforces that while the 0.3% extract initially provided significant protective effects, its prolonged efficacy may be limited. Nonetheless, the findings of the current study are in line with the decrease in LPO levels observed in the liver of seabass (*Dicentratchus labrax*) fed with a diet supplemented with 5% *Gracilaria* sp. and subjected to Phdp (*Photobacterium samselae* subsp. *piscicida*) infection [64]. Additionally, Thanigaivel et al. [65] found that the antioxidant response of *Oreochromis mossambicus* was improved against *Aeromonas* infection in fish fed with microencapsulated extracts of *Gracilaria foliifera* and *Sargassum longifolium*.

## 5. Conclusions

The present study provided valuable insights into the effects of dietary *L. digitata* supplementation, evaluating different inclusion levels and methods of employment on the performance and stress response of a high-value fish species under adverse environmental conditions, such as MHWs and disease outbreaks. Overall, the findings of this study demonstrate that supplementing aquafeeds with *L. digitata* maintained or even improved the growth performance and overall well-being of juvenile *S. aurata* under control temperature conditions. Incorporating *L. digitata* in either powdered (1.5%) or extract (0.3%) form significantly reduced the negative effects associated with MHWs and bacterial infections. However, given that over time the beneficial effects of the 0.3% extract diet ultimately aligned with those of the 1.5% powdered diet, the powdered form may represent a better cost-effective supplementation strategy, due to its lower processing requirements and wider availability, offering similar protective effects while reducing production costs. In summary, the results of this study indicate that *L. digitata* can be a valuable aquafeed supplement, enhancing both the performance and stress resilience in juvenile gilthead seabream. With the growing impacts of climate change, incorporating such functional feed additives presents a valuable strategy to strengthen the resilience, sustainability and robustness of aquaculture systems.

As a final remark, it is important to acknowledge that the present findings are influenced by the statistical power associated with the experimental design and should therefore be interpreted within this context and further validated under diverse experimental conditions.

## Figures and Tables

**Table 1:** Diet formulation and proximate composition of the four experimental diets. Abbreviations: CTR—control feed; 0.3P—feed supplemented with 0.3% of dried powdered *L. digitata*; 1.5P—feed supplemented with 1.5% of dried powdered *L. digitata*; 0.3E—feed supplemented with 0.3% of *L. digitata* extract.

Ingredients (%)	Experimental Diets			
	CTR	0.3P	1.5P	0.3E
Fishmeal Super Prime ^a^	17.0	17.0	17.0	17.0
Fish protein hydrolysate ^b^	2.0	2.0	2.0	2.0
Poultry meal ^c^	7.0	7.0	7.0	7.0
Soy protein concentrate ^d^	6.0	6.0	6.0	6.0
Wheat gluten ^e^	10.0	10.0	10.0	10.0
Corn gluten meal ^f^	11.0	11.0	11.0	11.0
Soybean meal (Hipro) ^g^	12.8	12.8	12.8	12.8
Wheat meal ^h^	12.0	11.7	10.5	11.7
Whole peas ^i^	6.0	6.0	6.0	6.0
Vitamin and mineral premix ^j^	1.0	1.0	1.0	1.0
Choline chloride 50% ^k^	0.2	0.2	0.2	0.2
Monoammonium phosphate ^l^	1.0	1.0	1.0	1.0
Fish oil ^m^	6.0	6.0	6.0	6.0
Salmon oil ^n^	8.0	8.0	8.0	8.0
*Laminaria digitata* powder ^o^	-	0.3	1.5	-
*Laminaria digitata* extract ^p^	-	-	-	0.3
Proximate composition (%)				
Crude protein	46.0	46.0	45.9	46.0
Crude fat	16.0	16.0	16.0	16.0
Fiber	1.4	1.4	1.4	1.4
Starch	13.5	13.3	12.5	13.3
Ash	7.4	7.4	7.7	7.4
Gross energy (MJ/kg feed)	20.5	20.5	20.4	20.5

^a^ Diamante, Pesquera Diamante, Peru (crude protein, CP: 66.3% dry matter, DM; crude fat, CF: 11.5% DM). ^b^ CPSP90, Sopropêche, France (CP: 82.6% DM; CF: 9.6% DM). ^c^ SAVINOR UTS, Portugal (CP: 67.4% DM; CF: 12.5% DM). ^d^ Soycomil P, ADM, The Netherlands (CP: 62.2% DM; CF: 0.7% DM). ^e^ VITAL, Roquette, France (CP: 80.4% DM; CF: 5.8% DM). ^f^ COPAM, Portugal (CP: 61.2% DM; CF: 5.2% DM). ^g^ Alphasoy 530, ABNeo AS, Denmark (CP: 52.9% DM; CF: 2.6% DM. ^h^ Molisur, Spain (CP: 11.7% DM; CF: 1.6% DM). ^i^ Ribeiro & Sousa, Portugal (CP: 19.6% DM; CF: 2.2% DM). ^j^ Premix for marine fish, PREMIX Lda, Portugal. Vitamins (IU or mg kg^−1^ diet): DL-alpha-tocopherol acetate, 100 mg; sodium menadione bisulphate, 25 mg; retinyl acetate, 20,000 IU; DL-cholecalciferol, 2000 IU; thiamine, 30 mg; riboflavin, 30 mg; pyridoxine, 20 mg; cyanocobalamin, 0.1 mg; nicotidin acid, 200 mg; folic acid, 15 mg; ascorbic acid, 1000 mg; inositol, 500 mg; biotin, 3 mg; calcium panthotenate, 100 mg; choline chloride, 1000 mg, betaine, 500 mg. Minerals (g or mg kg^−1^ diet): cobalt carbonate, 0.65 mg; copper sulfate, 9 mg; ferric sulfate, 6 mg; potassium iodide, 0.5 mg; manganese oxide, 9.6 mg; sodium selenite, 0.01 mg; zinc sulfate. 7.5 mg; sodium chloride, 400 mg; calcium carbonate, 1.86 g; excipient wheat middling’s. ^k^ ORFA, The Netherlands. ^l^ OMNISAL GmbH, Germany. ^m^ Sopropêche, France (CF: 98.1% DM). ^n^ Sopropêche, France (CF: 98.3% DM). ^o^ Parc Marin d’Iroise, Brittany, France. ^p^ Parc Marin d’Iroise, Brittany, France.

**Table 2:** Growth performance of juvenile *Sparus aurata* fed the experimental diets after 30 days of supplementation under control temperature conditions (T1), after 24 h after reaching the MHW peak temperature and *V. harveyi* challenge (T2), after 7 days of exposure to the MHW peak temperature alone (T3) and after 7 days of exposure to a combination of the MHW peak temperature and the *V. harveyi* challenge (T3.1) (mean ± SD; *n* = 6).

		Time	TL (cm)	W (g)	K	HSI (%)	SGR (% day ^−1^)	FCR
Supplementation + MHW conditions	CTR	T1	14.21 ± 0.64	47.21 ± 3.80	1.61 ± 0.10	1.11 ± 0.16 ^a^	1.42 ± 0.14 ^b^	0.38 ± 0.03 *
	0.3P	T1	14.38 ± 1.05	52.83 ± 6.82	1.61 ± 0.13	0.84 ± 0.08 ^c^	2.07 ± 0.31 ^a^*	0.34 ± 0.05 *
	1.5P	T1	14.42 ± 0.86	48.13 ± 2.74 *	1.61 ± 0.15	0.86 ± 0.10 ^bc^	1.59 ± 0.13 ^b^	0.37 ± 0.02 *
	0.3E	T1	14.64 ± 0.75	53.70 ± 6.97	1.66 ± 0.22	1.01 ± 0.12 ^ab^*	2.10 ± 0.36 ^a^*	0.34 ± 0.04 *
	CTR	T3	14.73 ± 0.61	53.78 ± 4.73	1.68 ± 0.32	1.10 ± 0.18 ^a^	1.13 ± 0.13 ^b^	0.51 ± 0.04 ^ab#^
	CTRHW	T3	15.38 ± 0.81	57.35 ± 5.41	1.50 ± 0.11	0.72 ± 0.05 ^b^	1.09 ± 0.16 ^b^	0.49 ± 0.05 ^ab#^
	0.3P	T3	15.26 ± 0.63	52.22 ± 2.90	1.53 ± 0.10	0.62 ± 0.04 ^b^	1.12 ± 0.09 ^b#^	0.55 ± 0.03 ^a#^
	1.5P	T3	15.72 ± 0.50	58.61 ± 3.87 ^#^	1.49 ± 0.17	0.74 ± 0.05 ^b^	1.45 ± 0.06 ^a^	0.46 ± 0.02 ^b#^
	0.3E	T3	15.29 ± 0.58	54.21 ± 4.61	1.50 ± 0.16	0.63 ± 0.08 ^b#^	1.12 ± 0.14 ^b#^	0.52 ± 0.04 ^a#^
MHW conditions + *V. harveyi* challenge	CTRHW	T2	14.96 ± 0.68	48.52 ± 2.62 ^b$^	1.48 ± 0.09	0.78 ± 0.06 ^b^	1.09 ± 0.09 ^b^	0.52 ± 0.03 ^a^
	CTRHW-PAT	T2	15.56 ± 0.73	58.24 ± 7.37 ^ab^	1.58 ± 0.12	1.15 ± 0.08 ^a^	1.91 ± 0.23 ^a$^	0.41 ± 0.15 ^ab^
	0.3P	T2	15.98 ± 0.73	58.29 ± 4.43 ^a^	1.52 ± 0.07 ^$^	1.11 ± 0.11 ^a^	1.66 ± 0.15 ^a$^	0.41 ± 0.04 ^b$^
	1.5P	T2	15.48 ± 0.81	55.92 ± 4.68 ^a^	1.61 ± 0.15	1.14 ± 0.08 ^a$^	1.65 ± 0.18 ^a^	0.43 ± 0.04 ^b^
	0.3E	T2	15.98 ± 0.71	61.42 ± 3.14 ^a^	1.51 ± 0.10	1.08 ± 0.10 ^a^	1.78 ± 0.09 ^a$^	0.41 ± 0.02 ^b^
	CTRHW	T3.1	15.38 ± 0.81	57.35 ± 5.41 ^/^	1.50 ± 0.11 ^ab^	0.72 ± 0.05 ^c^	1.09 ± 0.16 ^c^	0.49 ± 0.05 ^ab^
	CTRHW-PAT	T3.1	15.57 ± 0.59	56.28 ± 4.95	1.57 ± 0.12 ^a^	1.30 ± 0.18 ^a^	1.27 ± 0.09 ^bc/^	0.50 ± 0.04 ^ab^
	0.3P	T3.1	16.02 ± 0.55	54.37 ± 4.74	1.35 ± 0.06 ^b/^	0.93 ± 0.09 ^b^	1.15 ± 0.14 ^c/^	0.53 ± 0.04 ^a/^
	1.5P	T3.1	16.04 ± 0.48	61.39 ± 3.13	1.52 ± 0.13 ^ab^	0.92 ± 0.08 ^b/^	1.54 ± 0.08 ^a^	0.46 ± 0.02 ^b^
	0.3E	T3.1	15.59 ± 0.51	57.46 ± 4.19	1.55 ± 0.11 ^a^	0.98 ± 0.08 ^b^	1.39 ± 0.09 ^ab/^	0.47 ± 0.04 ^ab^

In each column, different letters denote significant differences (*p* < 0.05) between treatments at the same sampling time. Symbols * and ^#^ represent significant differences between sampling times T1 and T3 (Supplementation + MHW conditions), while symbols ^$^ and ^/^ stand for significant differences between sampling times T2 and T3 (MHW conditions + *V. harveyi* challenge) for the same treatment (*p* < 0.05). The absence of letters or symbols indicates no statistical difference. Abbreviations: CTR—control feed; CTRHW—control feed exposed to MHW conditions; CTRHW-PAT—control feed exposed to combination of MHW conditions and a *V. harveyi* challenge; 0.3P—feed supplemented with 0.3% of dried powdered *L. digitata*; 1.5P—feed supplemented with 1.5% of dried powdered *L. digitata*; 0.3E—feed supplemented with 0.3% of *L. digitata* extract; TL—total length; W—weight; K—Fulton’s condition index; HSI—hepatosomatic index; SGR—specific growth rate; FCR—feed conversion ratio.

**Table 3:** Plasma biochemistry values for juvenile *Sparus aurata* fed the experimental diets after 30 days of supplementation under control temperature conditions (T1), after 24 h after reaching the MHW peak temperature and *V. harveyi* challenge (T2), after 7 days of exposure to the MHW peak temperature alone (T3) and after 7 days of exposure to a combination of the MHW peak temperature and the *V. harveyi* challenge (T3.1) (mean ± SD; *n* = 5).

		Time	GLU (mg dL^−1^)	TP (g dL^−1^)	BUN (mg dL^−1^)	PHOS (mg dL^−1^)	ALT (U L^−1^)	CA (mg dL^−1^)	GLOB (g dL^−1^)	ALP (U L^−1^)	CHOL (mg dL^−1^)	AMY (U L^−1^)	Cortisol (ng mL^−1^)
Supplementation + MHW conditions	CTR	T1	80.33 ± 4.04 ^b^	3.15 ± 0.21 ^a^	7.73 ± 1.70	7.73 ± 0.64	22.00 ± 2.65 ^a^*	11.93 ± 0.35 ^b^	2.62 ± 0.24	233.67 ± 12.70 ^a^	164.33 ± 7.57	36.00 ± 9.42	46.84 ± 1.36
	0.3P	T1	110.67 ± 5.69 ^a^*	3.13 ± 0.34 ^a^	9.08 ± 1.72	9.06 ± 0.59	15.33 ± 0.58 ^b^	15.35 ± 0.72 ^a^*	2.48 ± 0.33	234.33 ± 6.81 ^a^	170.75 ± 39.85	25.75 ± 2.75	43.54 ± 5.21 *
	1.5P	T1	121.33 ± 7.23 ^a^	2.53 ± 0.05 ^b^	8.13 ± 1.65	7.92 ± 0.60	14.33 ± 1.53 ^b^	12.33 ± 0.28 ^b^*	2.20 ± 0.10	205.00 ± 9.54 ^b^*	148.50 ± 8.10	27.00 ± 4.08	52.40 ± 8.18
	0.3E	T1	121.00 ± 12.29 ^a^	2.55 ± 0.29 ^ab^*	8.13 ± 0.92	7.46 ± 0.77	19.00 ± 3.46 ^ab^*	11.38 ± 0.34 ^b^	2.24 ± 0.34	191.00 ± 7.00 ^b^	136.25 ± 32.57	25.00 ± 3.37	43.78 ± 5.48 *
	CTR	T3	94.67 ± 4.62 ^b^	2.85 ± 0.37 ^ab^	6.55 ± 1.06 ^ab^	6.74 ± 0.89	11.00 ± 1.00 ^ab#^	12.65 ± 0.87 ^ab^	2.26 ± 0.23	197.33 ± 21.83 ^b^	176.75 ± 18.82 ^ab^	32.25 ± 7.37	56.38 ± 2.60 ^b^
	CTRHW	T3	78.67 ± 9.87 ^b^	2.60 ± 0.24 ^b^	5.88 ± 0.57 ^b^	5.90 ± 0.45	10.33 ± 0.58 ^b^	11.10 ± 0.74 ^b^	2.34 ± 0.26	197.00 ± 6.24 ^b^	150.00 ± 13.29 ^b^	24.25 ± 5.56	61.56 ± 15.36 ^ab^
	0.3P	T3	130.67 ± 2.52 ^a#^	3.28 ± 0.05 ^a^	7.20 ± 0.29 ^a^	7.30 ± 0.70	14.00 ± 1.73 ^a^	13.85 ± 0.33 ^a#^	2.50 ± 0.14	222.00 ± 13.89 ^ab^	180.50 ± 21.46 ^ab^	19.75 ± 6.50	76.88 ± 6.77 ^a#^
	1.5P	T3	176.33 ± 41.19 ^a^	2.98 ± 0.22 ^ab^	8.33 ± 1.43 ^a^	7.44 ± 1.05	14.67 ± 3.21 ^a^	13.48 ± 0.71 ^a#^	2.32 ± 0.23	255.00 ± 16.09 ^a#^	175.75 ± 17.37 ^ab^	22.25 ± 6.65	62.62 ± 6.28 ^ab^
	0.3E	T3	133.33 ± 9.02 ^a^	3.23 ± 0.34 ^ab#^	6.28 ± 1.63 ^ab^	7.18 ± 1.46	10.17 ± 0.12 ^b#^	13.08 ± 0.97 ^a^	2.34 ± 0.13	225.67 ± 5.03 ^b^	201.00 ± 9.83 ^a^	20.50 ± 3.87	64.81 ± 6.71 ^ab#^
MHW conditions + *V. harveyi* challenge	CTRHW	T2	103.33 ± 13.01	2.75 ± 0.13	9.78 ± 1.60 ^a$^	7.28 ± 0.70 ^b^	22.33 ± 3.06 ^c^	11.13 ± 0.84 ^b^	2.44 ± 0.32	250.00 ± 18.19 ^b$^	128.50 ± 21.76	34.25 ± 9.32	55.06 ± 5.21 ^c^
	CTRHW-PAT	T2	84.33 ± 12.86 ^$^	3.05 ± 0.13	5.53 ± 0.64 ^bc^	11.68 ± 2.35 ^a$^	288.00 ± 30.81 ^a$^	11.13 ± 0.79 ^b^	2.24 ± 0.18	368.00 ± 44.00 ^a$^	120.25 ± 10.50	28.50 ± 4.80	92.68 ± 2.87 ^a^
	0.3P	T2	89.00 ± 3.46	3.28 ± 0.10	5.48 ± 0.56 ^bc^	10.78 ± 2.76 ^a^	367.00 ± 50.74 ^a$^	12.45 ± 0.66 ^ab^	2.20 ± 0.16	330.67 ± 28.54 ^a^	141.25 ± 10.11	22.25 ± 5.91	91.00 ± 6.50 ^a$^
	1.5P	T2	96.67 ± 4.04	2.98 ± 0.26	5.03 ± 0.05 ^c$^	10.06 ± 1.71 ^a^	172.67 ± 23.18 ^b$^	12.30 ± 0.42 ^b^	1.88 ± 0.16	338.00 ± 31.58 ^a$^	138.50 ± 15.78	31.25 ± 1.26	79.51 ± 2.57 ^b$^
	0.3E	T2	96.67 ± 7.02 ^$^	3.30 ± 0.42	6.30 ± 0.73 ^b^	11.80 ± 2.16 ^a$^	198.33 ± 45.80 ^b$^	14.28 ± 0.85 ^a^	1.94 ± 0.27	306.00 ± 41.07 ^a^	110.50 ± 23.59	25.00 ± 4.55	53.95 ± 1.43 ^c^
	CTRHW	T3.1	78.67 ± 9.87 ^c^	2.60 ± 0.24	5.88 ± 0.57 ^ab/^	5.90 ± 0.45 ^b^	10.33 ± 0.58 ^b^	11.10 ± 0.74 ^b^	2.34 ± 0.26	197.00 ± 6.24 ^c/^	150.00 ± 13.29 ^a^	24.25 ± 5.56 ^ab^	61.56 ± 15.36 ^ab^
	CTRHW-PAT	T3.1	133.33 ± 3.79 ^a/^	2.58 ± 0.44	5.95 ± 0.91 ^ab^	7.54 ± 0.88 ^a/^	10.03 ± 0.05 ^b/^	12.43 ± 0.95 ^ab^	1.80 ± 0.24	222.33 ± 4.16 ^b/^	119.25 ± 20.25 ^ab^	18.75 ± 4.86 ^b^	80.60 ± 10.91 ^a^
	0.3P	T3.1	105.33 ± 4.51 ^b^	2.93 ± 0.54	5.65 ± 0.79 ^ab^	7.48 ± 0.91 ^a^	40.33 ± 14.01 ^a/^	12.65 ± 1.37 ^ab^	1.88 ± 0.30	282.67 ± 18.50 ^a^	121.75 ± 20.66 ^ab^	39.25 ± 13.89 ^a^	50.14 ± 1.20 ^b/^
	1.5P	T3.1	98.67 ± 7.37 ^b^	2.85 ± 0.44	6.20 ± 0.45 ^a/^	7.22 ± 0.86 ^a^	10.09 ± 0.08 ^b/^	12.50 ± 0.73 ^b^	1.72 ± 0.40	206.67 ± 13.43 ^bc/^	117.00 ± 5.89 ^b^	17.75 ± 7.23 ^ab^	55.38 ± 4.04 ^b/^
	0.3E	T3.1	130.00 ± 13.53 ^a/^	3.43 ± 0.54	5.35 ± 0.26 ^b^	8.46 ± 0.94 ^a/^	10.07 ± 0.06 ^b/^	14.40 ± 0.67 ^a^	1.90 ± 0.35	234.33 ± 13.80 ^b^	137.75 ± 2.87 ^a^	19.00 ± 2.45 ^b^	51.04 ± 7.94 ^b^

In each column, different letters denote significant differences (*p* < 0.05) between treatments at the same sampling time. Symbols * and ^#^ represent significant differences between sampling times T1 and T3, while symbols ^$^ and ^/^ stand for significant differences between sampling times T2 and T3 for the same treatment (*p* < 0.05). The absence of letters or symbols indicates no statistical difference. Abbreviations: CTR—control feed; CTRHW—control feed exposed to MHW conditions; CTRHW-PAT—control feed exposed to combination of MHW conditions and a V. harveyi challenge; 0.3P—feed supplemented with 0.3% of dried powdered *L. digitata*; 1.5P—feed supplemented with 1.5% of dried powdered *L. digitata*; 0.3E—feed supplemented with 0.3% of *L. digitata* extract; GLU—glucose; TP—total protein; BUN—blood urea nitrogen; PHOS—phosphate; ALP—alkaline phosphatase; CA—calcium; GLOB—globulin; ALT—alanine aminotransferase; CHOL—cholesterol; AMY—amylase.

## Data Availability

Data are contained within the article or Supplementary Materials.

## Supplementary Materials

The following supporting information can be downloaded at: https://www.mdpi.com/article/10.3390/ani15131970/s1, Table S1: Two-way ANOVA *p*-values for the interaction between diet (CTR, 0.3P, 1.5P and 0.3E) and sampling time (T1, T2, T3 and T3.1) for each evaluated response variable.
