# Supplementary material for: Improving Farmed Juvenile Gilthead Seabream (Sparus aurata) Stress Response to Marine Heatwaves and Vibriosis Through Seaweed-Based Dietary Modulation
Source: Animals (Basel). 2025 Jul 4;15(13):1970. doi: 10.3390/ani15131970 (PMC12248664; doi:10.3390/ani15131970)
Supplement: Supplementary file 1 [file animals-15-01970-s001.zip › animals-3703349-supplementary.pdf]

animals

Table S1. Two-way ANOVA *p*-values for the interaction between diet (CTR, 0.3P, 1.5P and 0.3E) and sampling time (T1, T2, T3 and T3.1) for each evaluated response variable.

		**Two-way ANOVA (*p*-value)**	
		**Interaction Diet vs. Sampling point**	
		T1 vs. T3	T2 vs. T3.1
Growth performance indicators	TL	0.263	0.122
	W	0.002	< 0.001
	K	0.159	0.005
	HSI	< 0.001	< 0.001
	SGR	< 0.001	< 0.001
	FCR	< 0.001	< 0.001
Plasma metabolites	GLU	< 0.001	< 0.001
	TP	0.007	0.005
	BUN	0.415	< 0.001
	PHOS	0.250	0.003
	ALT	< 0.001	< 0.001
	CA	< 0.001	0.005
	GLOB	0.092	0.480
	ALP	< 0.001	0.007
	CHOL	0.130	0.010
	AMY	0.985	0.001
	Cortisol	0.007	< 0.001
Oxidative stress biomarkers (liver)	CAT	< 0.001	0.001
	GST	< 0.001	0.001
	SOD	0.162	0.772
	LPO	0.004	< 0.001

Abbreviations: TL – total length; W – weight; K - Fulton's condition index; HSI - hepatosomatic index; SGR - specific growth rate; FCR - feed conversion ratio; GLU – glucose; TP – total protein; BUN – blood urea nitrogen; PHOS – phosphate; ALT – alanine aminotransferase; CA – calcium; GLOB – globulin; ALP – alkaline phosphatase; CHOL – cholesterol; AMY – amylase: CAT – catalase; GST – glutathione S-transferase; SOD – superoxide dismutase; LPO – lipid peroxidation.
